# Comprehensive characterization of Fidgetin on tumor immune microenvironment evaluation and immunotherapy in human hepatocellular carcinoma

**DOI:** 10.18632/aging.205598

**Published:** 2024-02-27

**Authors:** Longju Qi, Shiyuan Chen, Zehua Liao, Mengjie Fan, Jiayi Zhang, Yuan Gao, Jiabao Shen, Yuyu Sun, Qinghua Wang

**Affiliations:** 1 Laboratory Animal Center, Medical School, Nantong University, Nantong, China; 2 Affiliated Nantong Hospital 3 of Nantong University, Nantong University, Nantong, China

**Keywords:** hepatocellular carcinoma, Fidgetin, immune checkpoint, prognosis, immune infiltration

## Abstract

Most cancers have a downregulation of Fidgetin (FIGN), which has been linked to tumor growth. However, there aren’t many papers that mention FIGN’s connection to hepatocellular carcinoma (HCC). Here, FIGN expression in HCC tissues was markedly reduced as compared to nearby normal liver tissues. According to univariate and multivariate Cox regression, it served as an independent predictor of survival outcomes. Patients with high levels of FIGN expression had a worse outcome. FIGN was shown to be engaged in immune-related pathways and to have a positive correlation with immunological score and immune cells according to KEGG pathway analysis. In HCC patients, FIGN was substantially linked with immunological checkpoints and the hot tumor state. Additionally, immunotherapy and chemotherapy showed a significant therapeutic response in HCC patients with low FIGN expression. This research revealed that FIGN expression was tightly related to hepatoma immunity and might be employed as a biomarker to predict patient prognosis and guide medication.

## INTRODUCTION

Primary liver cancer is the second most prevalent cause of cancer-related death and the sixth most frequent malignant tumor worldwide [1, 2]. Hepatocellular carcinoma (HCC), a tumor generated from the parenchymal cells of the livers, makes up around 80% of the most prevalent histology of liver cancer globally [1]. Asians are responsible for approximately 75% of liver tumor cases worldwide, while China accounts for more than 50% of all liver cancer cases. In Qidong City, China, the age-standardized rate (ASR) per 100,000 people is 77.5 [3]. Due to the liver’s sluggish sensitivity to pain and congestion due to the lack of innervated neurons, it is frequently diagnosed in the middle and late stages when there are no longer any operational indications [4]. Treatment for liver cancer places a significant financial burden on the patient’s family as well as the entire community due to its high morbidity and mortality [5]. Although many therapeutic approaches, including targeted therapy and immunotherapy, have been used to treat liver cancer, the survival rate is still quite poor [6]. Therefore, the need to investigate effective biomarkers for the early detection and prognosis of liver cancer is urgent.

The modulation of tumor immune infiltrates and immunotherapy, particularly in the treatment of hepatocellular carcinoma, is heavily influenced by the tumor microenvironment (TME) [7]. TME, which is characterized by an immunosuppressive milieu and tumor vasculature, is a dynamic network involving immunosuppressive cell subsets, inflammatory factors, and malignant cells [8]. Leaky arteries, central hypoxia, dysregulated ECM remodeling, and tumor-infiltrating immune cells, which supported hepatocarcinogenesis, dominated the complex cross-talk niche [9]. Numerous immune cells, such as tumor-infiltrating lymphocytes (TILs), tumor-associated macrophages (TAMs), tumor-associated neutrophils (TANs), and myeloid-derived suppressor cells (MDSCs), were shown to be recruited in the HCC TME [10]. Nevertheless, obstacles were discovered with immune checkpoint therapy, notably in solid tumors, which are typically divided into immunologically hot and cold tumors. Because fewer cytotoxic immune cells exist in cold tumors, there are lesser mutations present, and there are more immune suppressor cells, which can result in a worsening of the clinical outcome when immune checkpoint blockade (ICB) therapy is used [11, 12]. Although a few publications showed that it was possible to convert cold tumors into hot tumors, the ICB method was plagued by a low response rate and unfavorable side effects [13]. The precise mechanism behind the association between TME and ICB in HCC patients is still unknown. Therefore, it is crucial to understand the intricate mechanism underlying TME and ICB treatment in HCC because it will help identify responsive individuals and enhance clinical therapeutic approaches.

The protein known as Fidgetin (FIGN) is a type of conserved ATP-dependent enzyme that plays a crucial role in cutting microtubules [14, 15]. As a member of the AAA family of ATPases, FIGN always functions as a chaperone in coordinating the assembly and disassembly of macro-protein complexes and is thus involved in a wide range of biological processes, including microtubule dynamics, proteasome function, membrane fusion, peroxisome biogenesis, and vesicle-mediated transport [16, 17]. Previous studies demonstrated that FIGN cut microtubules more selectively in their labile domain than in the acetylated sections [18, 19]. By harboring bundles of labile domain-oriented microtubules, depleting FIGN increased axonal growth in fetal neurons, while lacking FIGN in adult rat dorsal root ganglion (DRG) neurons boosted axonal growth when confronted with an inhibitory environment both *in vitro* and *in vivo* [20, 21]. During the middle to late stages of gestation, the defective mouse embryo displayed vigorous FIGN expression [22]. A positive overexpression of FIGN has been observed in the nucleus of human HCC tissues [23]. Other publications inclined to ascribe FIGN as an oncogene in HCC and pancreatic cancer [24, 25]. As a prognostic and therapeutic target for patients with liver cancer, FIGN therefore had the potential to be a biomarker.

Due to its intricate structure and essential molecular roles in controlling cell biological developments. Its significance in liver cancer diagnosis, prognosis, and association with immune infiltration are yet unclear. In the recent investigation, it was discovered that hepatocellular carcinoma has downregulated FIGN. Furthermore, there was a correlation between increased FIGN expression and risk variables and unfavorable prognostic indices. Furthermore, the levels of immune infiltration for hepatocellular carcinoma were associated with the diagnostic and prognostic values of FIGN. These results noted that FIGN’s fundamental function as a possible biomarker for people with HCC.

## MATERIALS AND METHODS

### Tissue samples

From Affiliated Nantong Hospital 3 of Nantong University, 30 pairs of HCC samples and their corresponding normal liver tissues were collected.

### Data mining in the TCGA and GEO database

By logging in to the official websites https://portal.gdc.cancer.gov/ and https://xenabrowser.net/, respectively, datasets on the transcriptional expression of FIGN and the corresponding clinical data were obtained from the TCGA (369 primary liver carcinoma and 50 adjacent normal tissue) and UCSC database (n=374) [26]. The total number of cancer types included in this study was 21. For the sake of the subsequent study, the FPKM workflow type data from the RNA-seq method were transformed into TPM format and log2 transformation. The difference in FIGN expression between normal tissues and HCC was further examined using data from GEO (www.ncbi.nlm.nih.gov/geo) (GSE121248, n=107; GSE25097, n=517). GSE121248 included differential gene expression analysis between chronic hepatitis B induced HCC and adjacent Normal tissues using Affymetrix gene arrays. GSE25097 included transcriptome profiling of frozen tissues (tumor and non-tumor) in early-to-advanced HCC patients. Since the data were obtained from TCGA and GEO, there is no need for an ethics check.

### Functional enrichment analysis of FIGN gene co-expression network

The LinkedOmics database (http://www.linkedomics.org) examined the co-expression interaction network of FIGN in the TCGA-HCC dataset. In order to show the differences, rank gene maps and heatmaps were created using the Spearman correlation test. The rank coefficient was set as FDR<0.05. Additionally, the “ClusterProfiler” package of R was adopted to summary the Gene Ontology (GO) function and Kyoto Encyclopedia of Genes and Genomes (KEGG) pathway that contains the co-expressed genes. Pictures were visualized by “ggplot2” package.

### Tumor -immune system interaction database (TISIDB), tumor immune estimation resource (TIMER) database

The expression of FIGN and tumor-infiltrating lymphocytes (TILs) in various human cancers was assessed using the TISIDB database (http://cis.hku.hk/TISIDB/index.php). In order to assess the relationships between FIGN and TILs, Spearman’s test was performed. Additionally, the TIMER, TCGA, and GEPIA2 databases were used to examine the connection between immune infiltrating cells (B cells, CD8^+^ T cells, CD4^+^ T cells, macrophages, neutrophils, and dendritic cells) and FIGN expression in hepatocellular carcinoma. To examine the relationship between FIGN expression and overall survival (OS), disease-free interval (DFI), progression-free interval (PFI), and disease-specific survival (DSS) in HCC and the pan-cancer, data from UCSC-Xena were downloaded and processed using the R software’s “survival” and “forestplot” packages.

### Comprehensive analysis of TME in TCGA-HCC subgroups associated the expression of FIGN

To determine the stromal score for each sample, we employed the ESTIMATE method. The immune enrichment score was then calculated based on the 29 immune gene sets using the single sample gene set enrichment analysis (ssGSEA) function of the R package “GSVA” on the gene sets of “c2.cp.kegg.v7.4.symbols.gmt” from the MSigDB dataset. We uploaded the gene expression profiles of the HCC samples to the CIBERSORTx website (https://cibersortx.stanford.edu) using 1000 permutations in order to discover the immune infiltration characteristics of the samples.

### Tumor tissues clustering based on the hot tumor related gene markers

The tumors were divided into hot and cold tumors based on the gene markers identified in the previous publication, which included CCL5, CD8A, PDCD1, CD8B, CXCR3, CXCR4, CXCL9, CXCL10, CD4, CD3E, CD274, and CXCL11. To perform unsupervised clustering and offer quantifiable data to support the number of probable clusters within the RNA-seq dataset, the “ConsensusClusterPlus” R program was selected. Here, RNA-seq data from 374 HCC patients in the TCGA database were included in the study. The crucial detection settings included the previously mentioned 1000 repetitions, maximum assessed k of 20, and 80% item resampling.

### Immunotherapy analysis and chemotherapeutic response prediction

Immunophenoscore (IPS) was used to examine the data obtained from the TCIA website (https://tcia.at/home) in order to monitor the immunotherapy effects in the low- and high-score patients and assess the benefits and drawbacks of anti-CTLA-4 and anti-PD-L1 therapies. Based on the tumor’s immunogenicity and the effectiveness of the immunotherapy, the IPS scores ranged from 0 to 10. To compare the statistical differences and create the images, the “ggpubr” R tool was utilized.

The Genomics of Drug Sensitivity in Cancer (GDSC, https://www.cancerrxgene.org) was used to examine the effects of medications between low- and high-FIGN expression HCC patients in order to predict the reaction of commonly used drugs and possible small molecules. The half-maximal inhibitory concentration (IC50) was assessed by ridge regression, and the prediction accuracy was examined by 10-fold cross-validation based on the GDSC dataset. These effects were predicted using the default parameters of the “pRRophetic” R package. In addition, factors such tissue type of “allSolidtumours” and batch effect of “combat” were deleted, and duplicate gene expression was chosen as the mean value.

### Statistical analysis

Student’s t-test, Mann-Whitney U-test, and logistic regression analysis were employed to analyze differences in FIGN expression between the various clinical pathological features. The cutoff value was established using R software’s “pROC” tool. To examine the impact of FIGN on survival, Kaplan-Meier and log-rank tests were run using the “survminer” R package and the Kaplan-Meier website. Using TISIDB and TIMER, it was possible to compare the differences in FIGN expression and TILs between several groups. *P*<0.05 was regarded as statistically significant, and all data were shown as means with standard deviations.

### Availability of data and materials

Publicly available datasets were analyzed in this study. This data can be found at TCGA and GEO datasets (accession number: GSE121248, GSE25097) and in the Supplementary Material.

## RESULTS

### Expression pattern of FIGN based on pan-cancer analysis and in HCC patients

**Figure 1:**
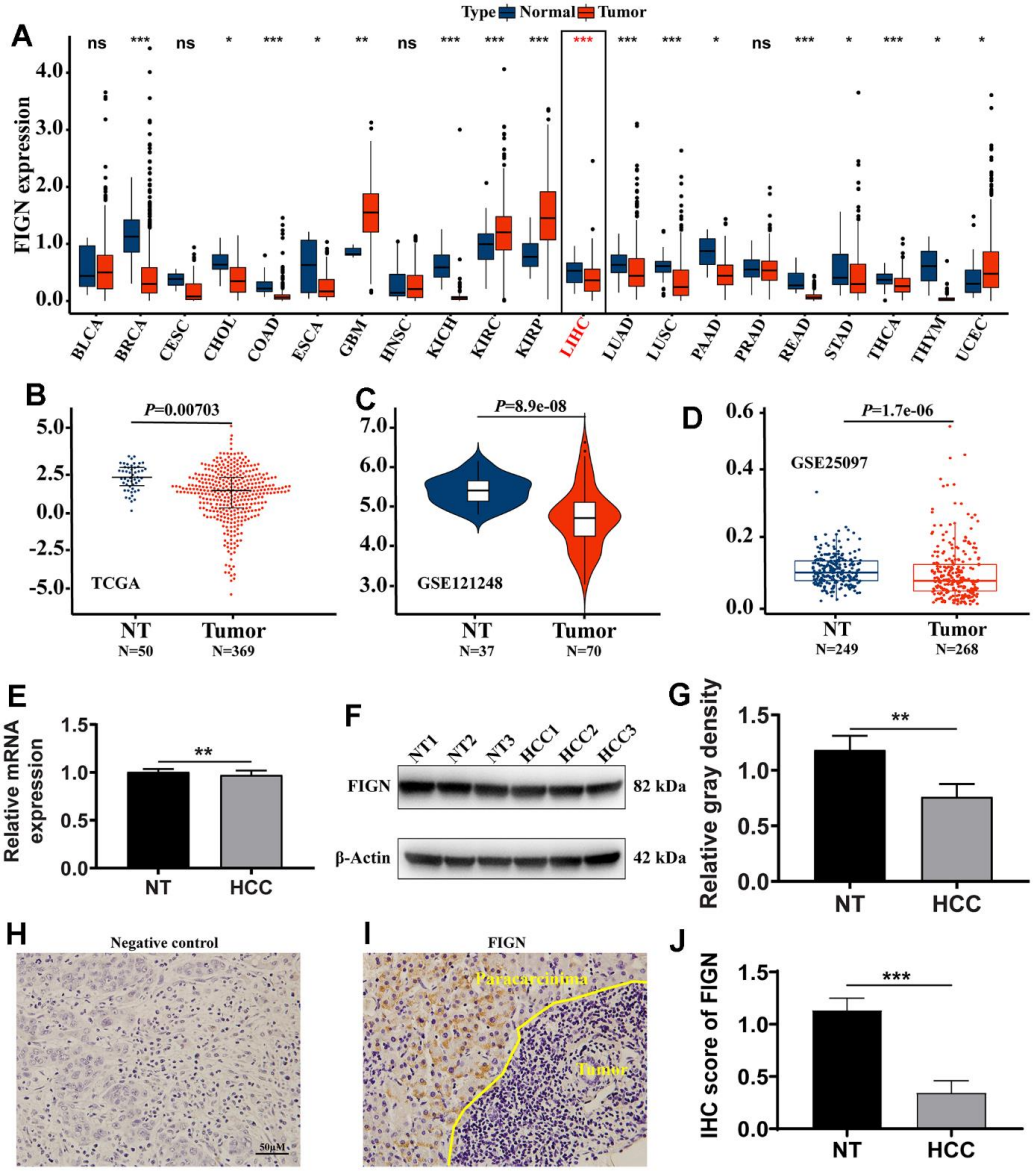
**The expression of FIGN in hepatocellular carcinoma and pan-carcinoma.** (**A**) The mRNA expression of *FIGN* was downregulated in 14 of 21 cancer types compared with normal tissues. Difference in expression of FIGN between HCC and normal tissues in TCGA data sets (**B**), GSE121248 (**C**) and GSE25097 (**D**). (**E**) Relative mRNA expression in HCC and paracarcinoma tissues. N=30. (**F**) Protein levels of FIGN in HCC and adjacent normal tissues. (**G**) Relative gray density analysis on bands of Figure F. (**H**–**I**) Immunohistochemistry assay to detect the expression of FIGN in HCC and paracarcinoma tissues. (**H**) was negative control. N=3, scale bar=100μM. (**J**) Difference in FIGN IHC score in HCC and matched paracarcinoma tissue. The two groups were compared using t-tests. *P < 0.05, **P < 0.01, ***P < 0.001, ns, no significance. BLCA, bladder urothelial carcinoma; BRCA, breast invasive carcinoma; CESC, cervical squamous cell carcinoma and endocervical adenocarcinoma; CHOL, cholangiocarcinoma; COAD, colon adenocarcinoma; ESCA, esophageal carcinoma; GBM, glioblastoma multiforme; HNSC, head and neck squamous cell carcinoma; KICH, kidney chromophobe; KIRC, kidney renal clear cell carcinoma; KIRP, kidney renal papillary cell carcinoma; LIHC, liver hepatocellular carcinoma; LUAD, lung adenocarcinoma; LUSC, lung squamous cell carcinoma; PAAD, pancreatic adenocarcinoma; PRAD, prostate adenocarcinoma; PCPG, pheochromocytoma and paraganglioma; READ, rectum adenocarcinoma; SARC, Sarcoma; SKCM, skin Cutaneous Melanoma; THCA, thyroid carcinoma; THYM, thymoma; STAD, stomach adenocarcinoma; UCEC, uterine corpus endometrial carcinoma.

Total of 21 cancer kinds were filtered out for further study in order to evaluate the transcriptional expression variations of FIGN among various cancer types. The results revealed that FIGN expression was decreased in 16 of the 21 cancer types as compared to the normal tissues. Additionally, compared to normal tissues, FIGN expression was greater in GBM, KIRC, KIRP, and UCEC (Figure 1A).

The transcriptional expression of FIGN was considerably downregulated in the primary tumor compared to paracarcinoma tissues, according to the results of the non-paired TCGA data analysis (Figure 1B, *P*=0.007). FIGN expression was not substantially different between patients of different ages (Supplementary Figure 1A), tumor stages (Supplementary Figure 1B), T stages (Supplementary Figure 1C), M stages (Supplementary Figure 1D), or N stages (Supplementary Figure 1E), but FIGN expression was significantly different between male and female patients (Supplementary Figure 1F, *P*<0.05). In Supplementary Table 1, the clinicopathological traits were described in depth. In addition, data sets from GSE121248 and GSE25097 revealed that FIGN mRNA expression was lower in tumors compared to normal tissues (Figure 1C, *P*=8.9E-08 and Figure 1D, *P*=1.7E-06).

Notably, FIGN’s mRNA expression was considerably lower in clinical HCC samples than it was in paracarcinoma tissues (Figure 1E, *P*<0.01) When compared to the corresponding normal tissues, FIGN protein expression was reduced in the main tumor (Figure 1F, 1G, *P*<0.01). FIGN expression in the tumors was shown to be prone by immunohistochemistry staining on HCC liver sections (Figure 1H–1J, *P*<0.001). Additionally, histological analysis provided in HPA demonstrated that FIGN protein was downregulated in malignancies (Supplementary Figure 1G). The information gathered showed that FIGN expression in hepatocellular carcinoma tissues was lowered on both the mRNA and protein levels.

### The prognostic value of FIGN in hepatocellular carcinoma

**Figure 2:**
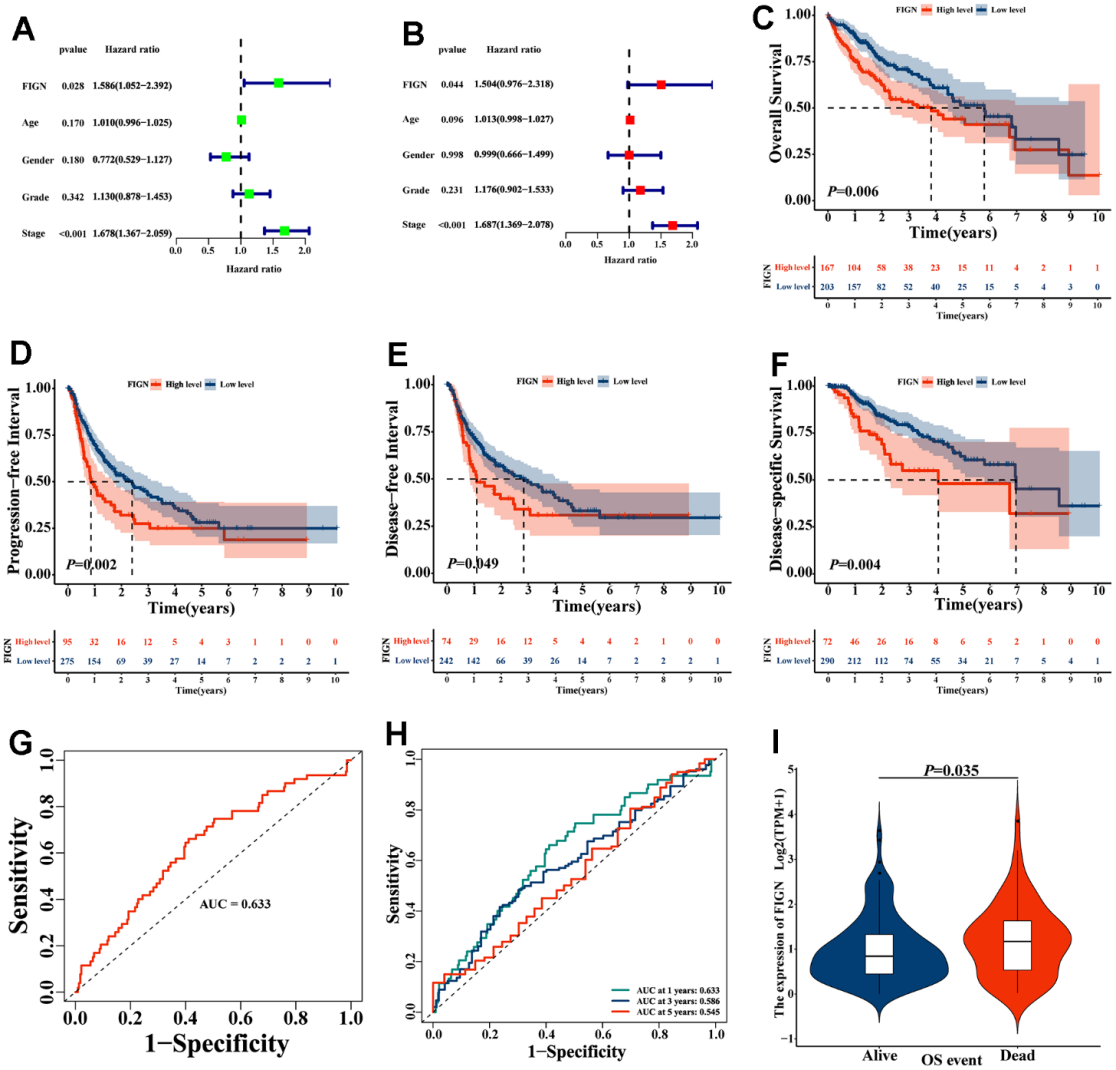
**The prognostic value of FIGN in hepatocellular carcinoma.** (**A**) Univariate Cox regression analysis of FIGN with age, gender, grade and stage. (**B**) Multivariate Cox regression analysis of FIGN with age, gender, grade and stage. Kaplan-Meier survival analysis stratified by FIGN expression in HCC patients and illustrated as overall survival (OS) (**C**), progression-free interval (PFI) survival (**D**) disease-free interval (DFI) survival (**E**) and disease-specific interval survival (DSS) (**F**). (**G**) ROC curve to predict sensitivity and specificity of survival based on FIGN expression in HCC patients. (**H**) ROC curve for predicting the sensitivity and specificity of 1-, 3-. and 5-year survival based on FIGN expression in HCC patients. (**I**) OS event.

The TCGA dataset underwent Cox regression analysis in order to further examine the prognostic value of FIGN. In both the univariate Cox regression analysis (Figure 2A) and the multivariate Cox regression analysis, the findings demonstrated that FIGN was strongly linked with the OS (Figure 2B). High FIGN expression generally predicted a poor prognosis compared to low-FIGN patients, especially in OS (*P*=0.006, Figure 2C), PFI (*P*=0.002, Figure 2D), DFI (*P*=0.049, Figure 2E), and DSS (*P*=0.004, Figure 2F). The clinical reliability of FIGN was investigated using a ROC curve, and the AUC value of 0.633 demonstrated significant sensitivity and specificity in HCC detection (Figure 2G). Additionally, the AUC values for the 1-year, 3-year, and 5-year OS were, respectively, 0.633, 0.586, and 0.545. (Figure 2H). According to the box plot (*P*=0.035, Figure 2I), individuals with high FIGN have considerably greater fatality rates than those with low FIGN. These findings suggested that FIGN might be a viable biomarker for HCC therapy.

### Enrichment analysis of FIGN gene co-expression network in HCC

To examine the co-expressed protein-coding genes connected to the expression of FIGN in the TCGA database’s HCC data sets. In total, 6635 genes with negative correlations and 13286 genes with positive correlations were presented in Supplementary Figure 2A. Heat maps were used to display the top 50 genes that favorably (Supplementary Figure 2B) and negatively (Supplementary Figure 2C) associated with FIGN expression. The GO function and KEGG pathway enrichment analyses included the top 200 genes that were positively co-expressed with FIGN. Top 10 bubble plots for BP, CC, and MF were displayed (Supplementary Figure 2D–2F). According to KEGG pathway analysis, FIGN co-expression genes were primarily enlisted in immune-related pathways (Supplementary Figure 2G). The findings in this study suggested that FIGN may contribute to an immune-related response in HCC patients.

**Figure 3:**
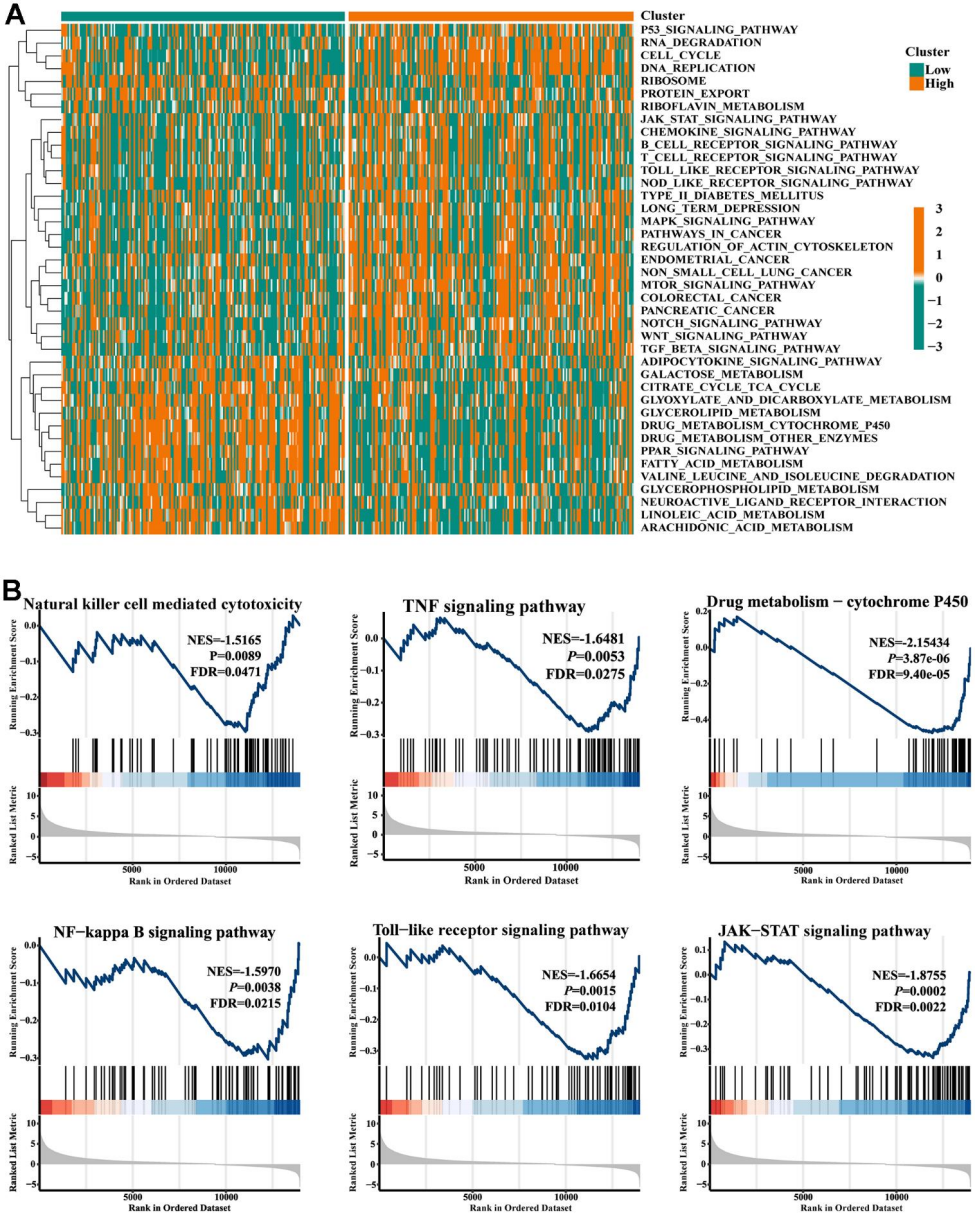
**Gene set enrichment analysis.** (**A**) Heat map showed the KEGG pathways differences between low- and high-FIGN expression patients. (**B**) Pathways enriched in the Nature killer cell cytotoxicity, TNF signaling, Drug metabolism-P450, NF-kappa B signaling, Toll-like receptor signaling, and JAK-STAT signaling.

The KEGG pathway variations between the high- and low-FIGN patients were then evaluated by GSVA and depicted in a heat map (Figure 3A). Drug metabolism pathways tended to be enriched in the low-FIGN group, but immune-related pathways, such as the JAK-STAT signaling pathway, Chemokine signaling pathway, and B cell receptor signaling pathway, etc., were significantly enriched in the high-FIGN group. GSEA was used to enrich the gene sets in order to examine the potential role of the FIGN gene. A total of 2718 gene sets were identified, including the TNF signaling pathway, NF-kappa B signaling pathway, Toll-like receptor signaling pathway, JAK-STAT signaling pathway, and Nature killer cell mediated cytotoxicity (FDR=0.047, *P*=8.9e-03), as well as drug metabolism-cytochrome P450 (FDR=9.4e-05, *P*=3.9e-06) (Figure 3B). According to the data presented above, FIGNinteracting genes were primarily involved in the control of immunity and drug metabolism.

### Correlation between FIGN and tumor immune infiltration cells

**Figure 4:**
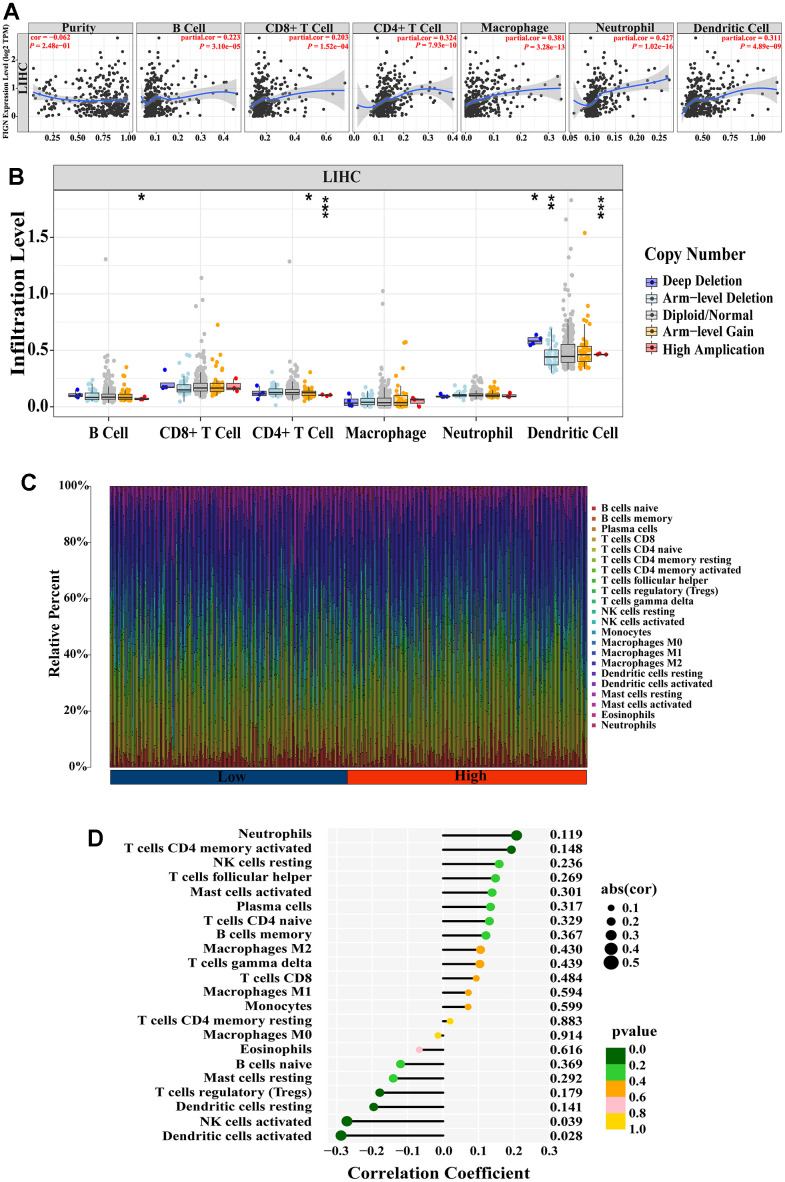
**Correlation between FIGN expression and immune cell infiltration in HCC patients.** (**A**) Correlation between the expression of FIGN and immune infiltrating cells in HCC. (**B**) Association between FIGN copy number variation (CNV) and B cell, CD4^+^ T cell, macrophages, neutrophils and dendritic cell in HC Comparison between multiple groups using analysis of variance. C. (**C**) Immune landscape variations between low- or high- FIGN expression groups based on CIBERSORT algorithms. (**D**) Correlation among infiltration levels of 22 immune cell types and FIGN expression profiles by Spearman’s analysis. *P < 0.05, **P < 0.01, ***P < 0.001, ns, no significance.

The association between FIGN expression and immune penetrating cells in HCC was discovered using the TIMER database. Results showed that FIGN expression was positively correlated with B cell (R=0.223, *P*=3.10e-05), CD8^+^ T cell (R=0.203, *P*=1.52e-04), CD4^+^ T cell (R=0.324, *P*=7.93e-10), macrophage (R=0.381, *P*=3.28e-13), neutrophil (R=0.427, *P*=1.02-16), and dendritic cell (R=0.311, *P*=4.89e-09), while negatively correlated with tumor purity (R=-0.062, *P*=2.48e-01) (Figure 4A). Additionally, B cell, CD4^+^ T cell, and dendritic cell infiltration levels were impacted by FIGN CNV (Copy Number Variation) (Figure 4B).

To comprehensively analyze the landscape of the immune microenvironment, CIBERSORTx was taken to examine the infiltration situation of 22 immune cells. Figure 4C shows the immunological characterization based on TCGA-LIHC samples. Additionally, Spearman’s analysis revealed a link between FIGN expression profiles and the infiltration levels of 22 immune cell types (Figure 4D). Neutrophils and activated CD4 memory T cells were the top two immune cells that positively linked with FIGN, while activated dendritic cell and NK cells were the top two immune cells that negatively correlated with FIGN. In conclusion, FIGN showed some relationship with immune invading cells.

**Figure 5:**
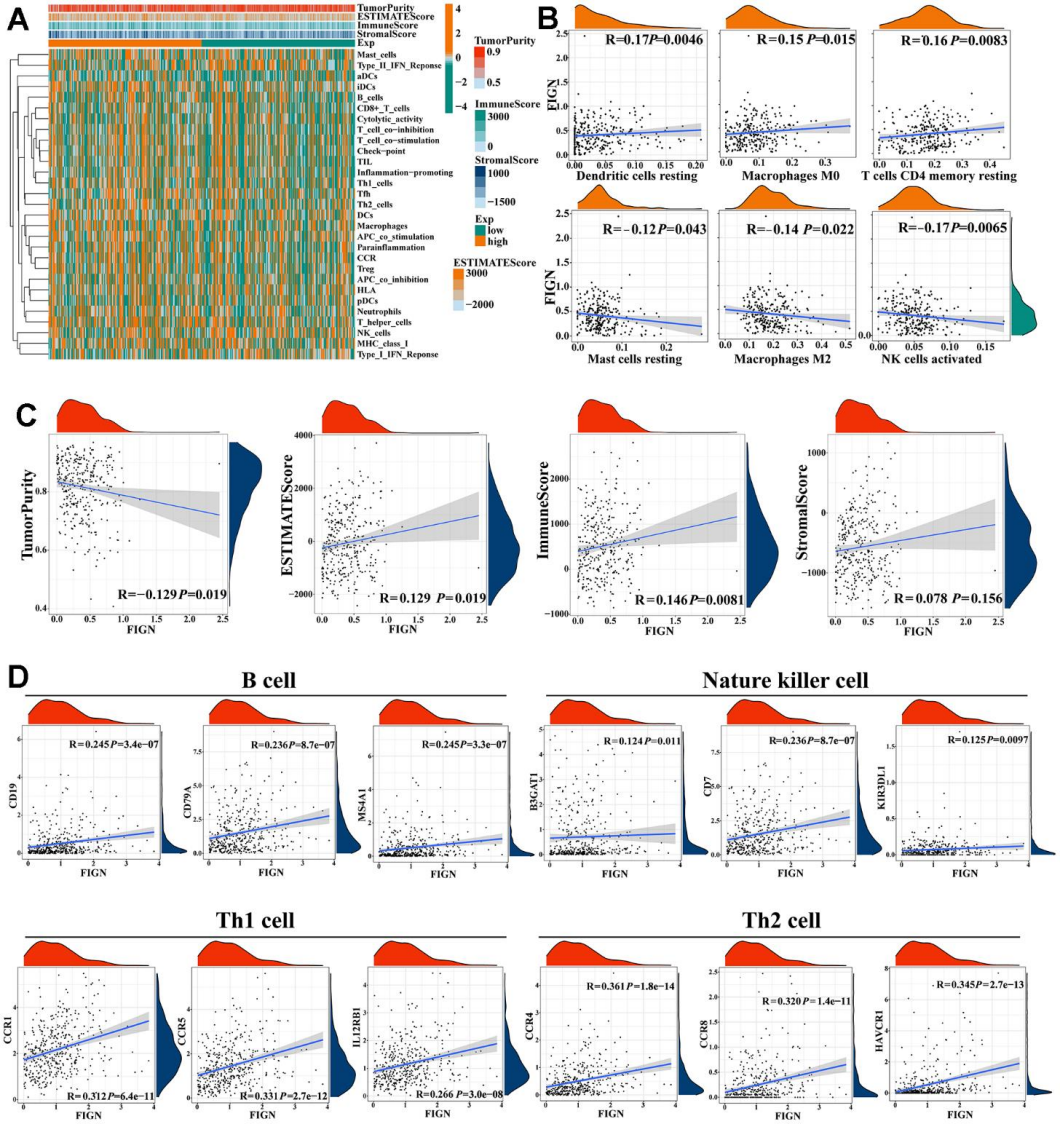
**Correlations of *FIGN* expression with immunotherapy related genes in HCC.** (**A**) Expression patterns of 29 immune cell subtypes in TumorPurity, ESTIMATEScore, ImmuneScore, and StromalScore between high- and low-FIGN expression groups in HCC tumor samples. (**B**) Correlation between FIGN expression and dendritic cells resting, macrophage M0, T cell CD4 memory resting, mast cell resting, macrophages M2 and NK cell activated. (**C**) Correlation between FIGN expression and TumorPurity, ESTIMATEScore, ImmuneScore, StromalScore. (**D**) Correlation between FIGN and immune cell markers include CD19, CD79A and MS4A1 of B cell; B3GAT1, CD7 and KIR3DL1 of nature killer cell; CCR1, CCR5 and IL12RB1 of Th1 cell; CCR4, CCR8 and HAVCR1 of Th2 cell.

The stromal score and immune activity of all samples have been shown by heatmap in order to compare the profiling of stromal cells and immune cells in the TME, which had a significant impact on tumor development, therapeutic effects, and clinical outcomes (Figure 5A). FIGN expression demonstrated a connection with tumor immune cell infiltration (Figure 5B). Additionally, FIGN expression illustrated positive correlations with ESTIMATEScore, ImmuneScore, and StromalScore while presenting negative correlations with TumorPurity, indicating its function in antitumor immunity (Figure 5C). Data sets from the GEPIA2, TIMER, and TCGA-LIHC databases were taken to assess the link between FIGN and distinct immune infiltrating cells in HCC by examining the association between FIGN and immunological markers of the immune cells (Supplementary Table 2). Results from the three databases showed a correlation between FIGN expression and the genes for B cells, natural killer cells, Th1 cells, and Th2 cells (Figure 5D). Together, FIGN may play a significant role in the control of tumor TME and immunity.

The enrichment scores of various immune cell subpopulations, associated functions, and linked pathways were measured using ssGSEA in order to further investigate the relationship between FIGN and immunological state. We discovered that the high-FIGN group had considerably higher levels of the components of the antigen presentation pathway in the TCGA cohort, including aDCs, iDCs, pDCs, APC co-inhibition, HLA, and MHC class I. (Supplementary Figure 3A). Additionally, the high-FIGN group had greater proportions of Tfh cells, Treg cells, Th1 cells, Th2 cells, T cell co-stimulation, and T cell co-inhibition. Furthermore, the high-FIGN patients had higher scores for CCR, check-point, macrophages, neutrophils, and inflammation-promoting activity, while type II IFN response activity was the exact reverse (Supplementary Figure 3B). In the TCGA, the low-FIGN group had a greater enrichment of immune cells, particularly anti-tumor immune cells (using the TIMER, CIBERSORT, CIBERSORT-ABS, QUANTISEQ, MCPCOUNTER, XCELL, and EPIC algorithms) (Supplementary Figure 3C). According to several platforms, the immune bubble diagram demonstrated the affiliation of various immune cells with FIGN expression (Supplementary Figure 3D). B cells and cancer-associated fibroblast (CAFs) were associated with the high-FIGN expression patients. Totally, FIGN were correlated with immune infiltrating cells and thus affected the TME.

### FIGN was correlated with immune checkpoints and enhanced immunotherapy effects

**Figure 6:**
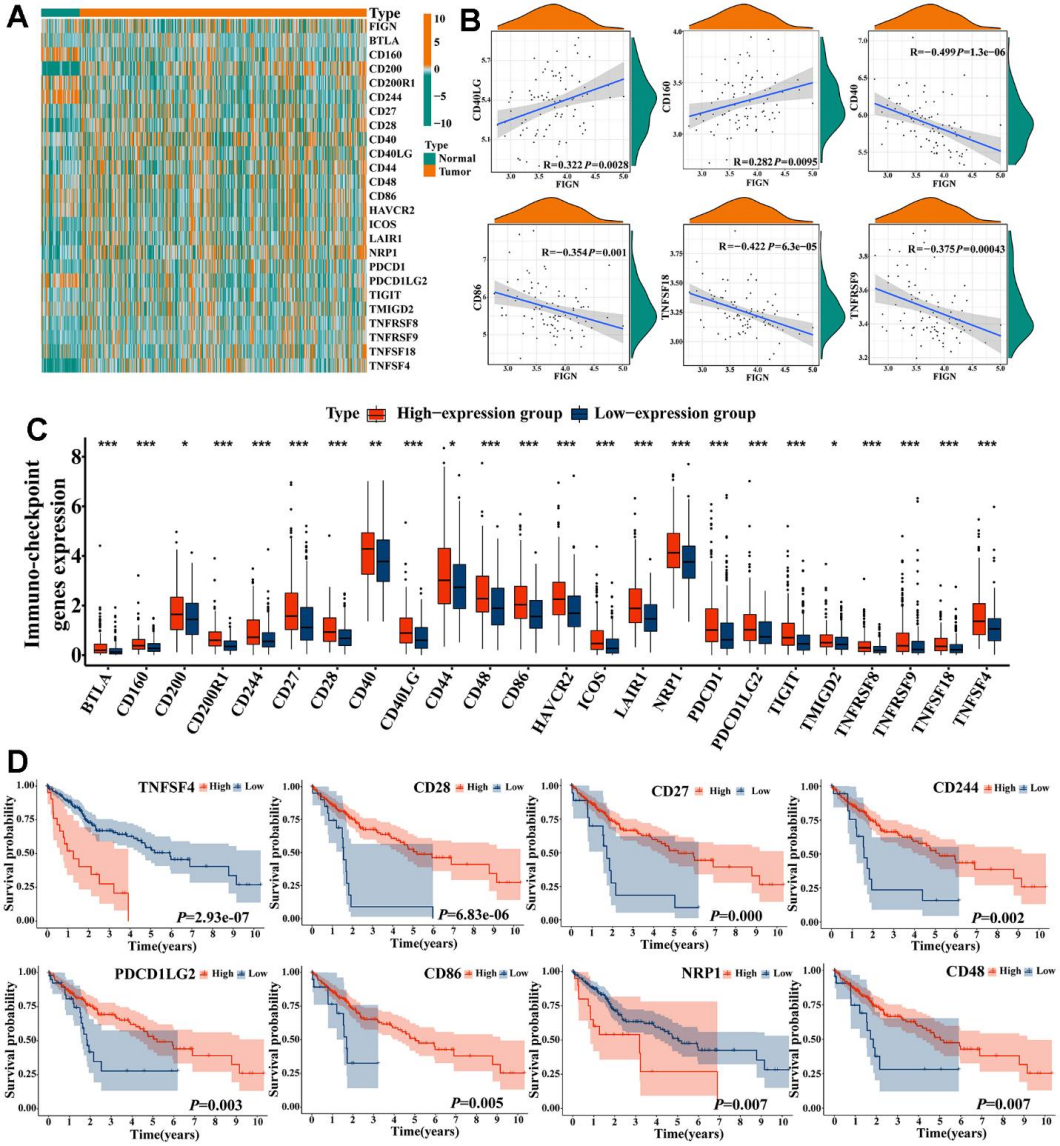
**Correlations of *FIGN* expression with immune checkpoint genes in HCC.** (**A**) Heatmap demonstrated the differences of FIGN and immune checkpoint genes. (**B**) Correlations between FIGN and the immunotherapy related genes expression, including CD40LG, CD160, CD40, CD86, TNFSF18 and TNFRSF9. (**C**) The differential expression of immunotherapy related genes between high- and low-FIGN expression groups in HCC samples. (**D**) Kaplan-Meier plot of TNFSF4, CD28, CD27, CD244, PDCD1LG2, CD86, NRP1 and CD48. The two groups were compared using t-tests. *P < 0.05, **P < 0.01, ***P < 0.001, ns, no significance.

The effects of immunotherapy mostly depended on the activation or inhibition of immune checkpoint gene activity. 24 immune checkpoint genes were identified in heatmap analysis to compare the expression patterns of immune checkpoints in normal and malignant tissues in HCC patients (Figure 6A). FIGN expression was positively connected with the expression of CD40LG (R=0.322, *P*=2.8e-03) and CD160 (R=0.282, *P*=9.5e-03), but its expression was negatively correlated with the expression of CD40 (R=-0.499, *P*=1.3e-06), CD86 (R=-0.354, *P*=0.001), TNFSF18 (R=-0.422, *P*=6.3e-05), and TNFRSF9 (R=-0.375, *P*=4.3e-04) (Figure 6B).

Between high- and low-FIGN groups, the expression of immune-checkpoint genes was compared (Figure 6C). High expression of CD28 (*P*=6.83e-06), CD27 (*P*=0.000), CD244 (*P*=0.002), PDCD1LG2 (*P*=0.003), CD86 (*P*=0.005), and CD48 (*P*=0.007) was shown in a Kaplan-Meier plot to be substantially linked with a better prognosis for HCC, while high expression of TNFSF4 (*P*=2.93e-07) and NRP1 (*P*=0.007) illustrated opposite survival outcome (Figure 6D). These findings suggested that FIGN may be linked to the HCC immunotherapy checkpoints.

In order to classify patients with qualitatively different hot tumors and cold tumors based on the expression of 12 hot tumor signature genes, the ConsensusClusterPlus R package was used [11, 12]. The results showed that K=3 appeared to be the best option for dividing the entire cohort into subtypes A (n=137, cold), B (n=90, hot), and C (n=197, non-response) (Supplementary Figure 4A–4D). FIGN expression was significantly higher in hot tumors than in cool tumors (*P*=3.5e-07, Supplementary Figure 4E), suggesting complex aspects should be taken into account when evaluating the effectiveness of immunotherapy, particularly in clinical trials. Thus, the findings above suggested that FIGN may have a role in the response to immunotherapy. Based on the TCGA RNA-seq data cohort, the association of FIGN with the predictors of response to immunotherapy was established in order to further investigate the therapeutic effects of FIGN. The findings showed that FIGN was significantly linked to the chosen parameters (Supplementary Figure 4F). Additionally, the expression of immunotherapy checkpoints in hot, cold, and non-responsive cancers varied significantly (Supplementary Figure 4G). The information gathered here positively showed that FIGN may have boosted therapeutic effectiveness in the treatment of HCC.

### Expression of FIGN predicted sensitivity of HCC patients to clinical immunotherapy and chemotherapy

**Figure 7:**
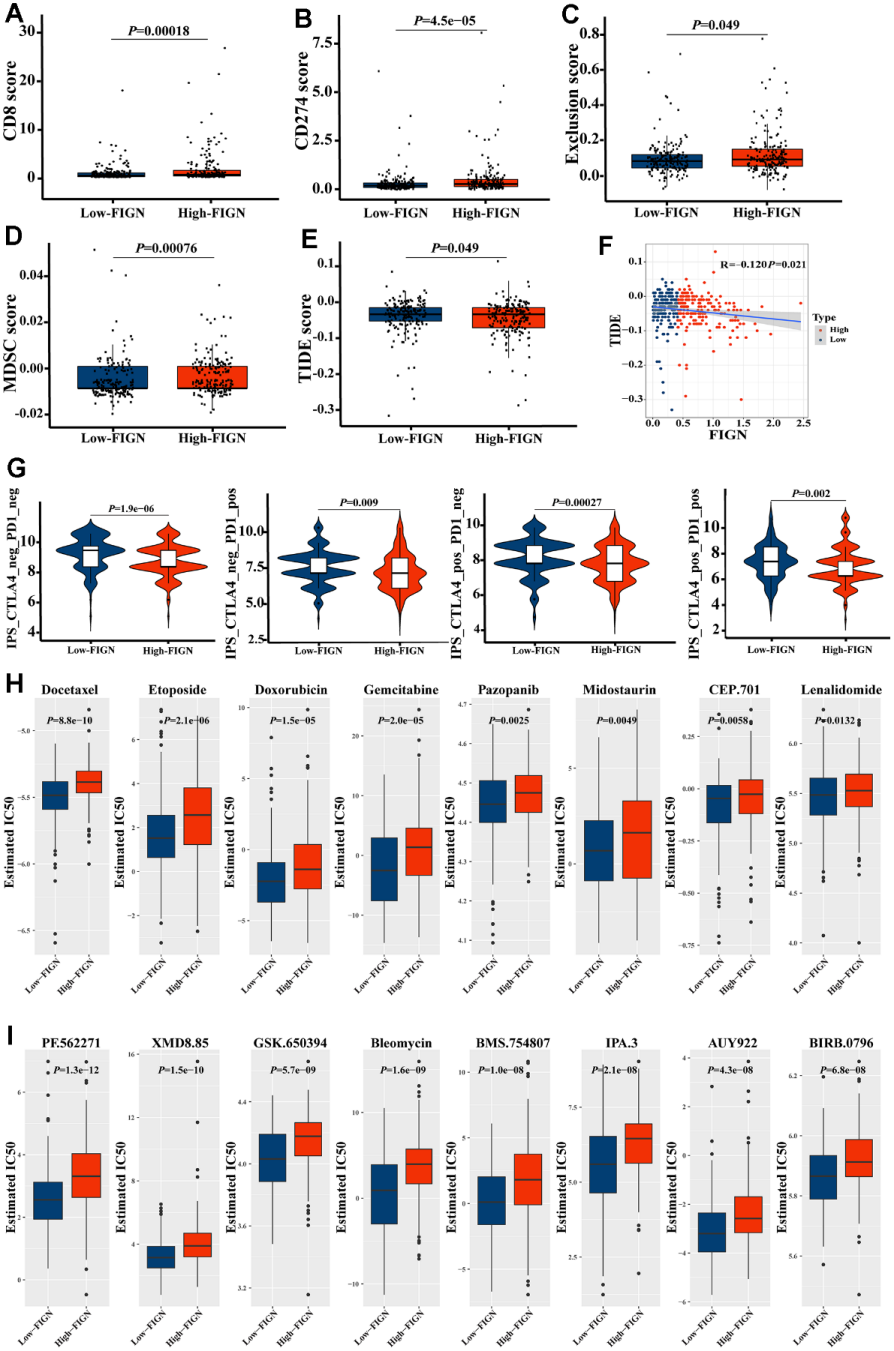
**FIGN expression predicted the responsiveness of HCC to immunotherapy, chemotherapy, and potential small-molecule compounds.** Box-plots show the differences in CD8 (**A**) and CD274 (**B**) score between the low- and high-FIGN expression groups. Box-plots show the differences in Exclusion score (**C**), MDSC score (**D**), and TIDE score (**E**) between the low- and high-FIGN expression groups. (**F**) Correlation between TIDE score and FIGN expression. (**G**) The association between IPS and the expression of FIGN based on TCIA database. (**H**) Relationships between FIGN expression and chemotherapeutic sensitivity of HCC. (**I**) Potential small-molecule compounds might function in HCC treatment. The two groups were compared using t-tests. *P < 0.05, **P < 0.01, ***P < 0.001, ns, no significance.

In the present study, TIDE score was utilized to further investigate the relevance of FIGN in predicting the immunotherapy response of HCC patients. As a consequence, patients with high FIGN had higher CD8 scores (Figure 7A) and CD274 scores (Figure 7B) than patients with low FIGN. Additionally, individuals with high levels of FIGN expression had higher Exclusion scores (Figure 7C), MDSC scores (Figure 7D), and TIDE scores (Figure 7E) than patients with low levels of FIGN expression. Additionally, TIDE and FIGN were found to be negatively correlated (Figure 7F). According to the data above, immunotherapy had a negative effect on patients who expressed high levels of FIGN.

Consistently, we investigated the CTLA-/PD-1 inhibitor response to ICI therapy further. The patients in the low-FIGN group had a substantial therapeutic response in HCC patients (Figure 7G). Next, we investigated the relationship between FIGN expression levels and drug sensitivity in HCC patients. Interestingly, we found that the IC50 values of Docetaxel, Etoposide, Doxorubicin, Gemcitabine, Pazopanib, Midostaurin, CEP.701 and Lenalidomide were higher in patients with high FIGN expression (Figure 7H). Furthermore, the IC50 values of PF.562271, XMD8.85, GSK.650394, Bleomycin, BMS.754807, IPA.3, AUY922 and BIRB.0796 were higher in patients with high FIGN expression (Figure 7I). Taken together, results here suggested that the expression of FIGN was associated with drug susceptibility.

## DISCUSSION

HCC is one of the most leading causes of cancerous mortality worldwide for its malignant aggressiveness and terrible prognosis [27]. Plenty of new developed strategies have been enrolled in treating HCC patients, the prognosis was still no obvious improvement [28]. To conquer this disease, extra efforts should be paid on specifying the underlying molecular mechanism, clarifying the fine progression of pathogenesis and unearthing promising biomarkers for diagnosis and prognosis. Less reports were focused on the expression and function of *FIGN* in HCC progression and prognosis, especially in TME and immune therapy [14, 23].

Fidgetin (FIGN) is reported to act as a conserved ATP-dependent enzyme and play important roles in regulating the homeostasis of microtubules by severing the labile domain [14, 18]. However, the function of FIGN in cancer development and treatment was not intensively studied. FIGN was positively overexpressed in the nucleus of human HCC tissues, according to earlier investigations [22, 23]. The scientists discovered that FIGN’s mRNA expression was around 1.8 times greater in HCC tissues than it was in the matched healthy liver. Additionally, as compared to nearby healthy tissues, HCC had a 2.1-fold increase in FIGN protein expression. The RNA-Seq data from 371 human HCCs in TCGA, however, revealed that the expression of FIGN mRNA in HCC was considerably lower than that of the nearby healthy tissues. Only 24 HCC and paired nearby normal liver tissues were used in the analysis, suggesting that the discrepancies in expression patterns may be due to sample selection.

The expression of FIGN was also low in 149 tumors (69%) and high in 67 tumors (31.0%), according to the clinicopathological features of the 216 individuals with HCC included in their study. Additionally, in another analysis, investigators stained a panel of 100 human HCC samples with FIGN and assessed four independent core biopsies from each patient’s tumor and normal surrounding liver [24]. Only 15% of human HCCs were found to express FIGN protein strongly, at levels noticeably greater than the matching normal tissue [24]. Moreover, Riordan et al. reported that analysis based on the RNA-Seq data from 371 human HCCs in the TCGA indicated only 15% of human HCCs overexpressed FIGN. These findings to a certain degree suggested that FIGN expression was downregulated in majority of HCC samples and exhibited certain roles in acting as a potential biomarker to differentiate HCC from normal liver tissues.

Tumor-infiltrating lymphocytes (TILs), a recently discovered prognostic biomarker, are linked to increased survival rates and higher rates of response to neoadjuvant therapy and immunotherapy in cancer patients [29, 30]. TILs frequently contain T cells, B cells, and natural killer (NK) cells, which are one of the exemplary elements of the host anticancer immune responses [31, 32]. The international TILs Working Group’s standards included a standardized TIL scoring system, and the average TIL score based on the entire stromal surface as opposed to hotspots showed significant clinical relevance in cancer detection [33, 34]. The role of FIGN in controlling immunological response, however, has not yet been documented. According to the results of the current study, FIGN has a preliminary role in pro-tumor immunity, including natural killer cell-mediated cytotoxicity, TNF signaling, NF-kappa B signaling, Toll-like receptor signaling, and JAK-STAT signaling. Further research revealed a substantial correlation between FIGN and immunological infiltrates in the TME, including dendritic cells resting, macrophages M0, T cells CD4 memory resting, mast cells resting, macrophages M2, and NK cells activated, etc. According to the TIMER database, FIGN expression consistently showed a positive correlation with B cells, CD8^+^ T cells, CD4^+^ T cells, macrophages, neutrophils, and dendritic cells. In more detail, FIGN expression demonstrated a positive correlation with the marker genes for Th1 and Th2 cells, nature killer cells, and B cells based on the TCGA, GEPIA, and TIMER data sets. Additionally, there was a strong correlation between FIGN CNV and the amounts of B cells, CD4^+^ T cells, and dendritic cells invasion. Results here indicated that FIGN participated in the immune response to the TME of HCC. The CIBERSORT tools were taken to sort out the proportion of 22 tumor immune cells in HCC, and significant differences were observed in regulatory T cells, gamma delta T cells, and monocytes with different expression levels of FIGN. These results suggest that FIGN played an important role in the immune regulation of HCC. Moreover, FIGN was found to augment the efficacy of immunotherapy for HCC. Expression of immune checkpoint genes was remarkably enhanced in the high-FIGN group compared with low-FIGN group. However, the expression pattern of checkpoints showed no obvious difference between normal and tumor tissues. Together, other factors underlying should be discovered to explain it. Immunologically hot tumors and cold tumors were classified based on the response or refractory to immunotherapy [11, 35]. FIGN was found to be increased in hot tumors and positively correlated with the immune checkpoint genes that suggested the potential function of FIGN in regulating the response of immunotherapy. Finally, the roles of FIGN in predicting chemotherapy response in HCC patients were reported in the current study and results demonstrated that patients with high FIGN expression exhibited certain resistance to treatment of Docetaxel, Etoposide, Doxorubicin, Gemcitabine, Pazopanib, Midostaurin, CEP.701 and Lenalidomide, Similar outcomes were predicted in small molecules as PF.562271, XMD8.85, GSK.650394, Bleomycin, BMS.754807, IPA.3, AUY922 and BIRB.0796. The significant differences between the HCC patients distinguished by expression of FIGN regarded its role of predicting effectiveness in potential drugs choice for the patients as positive.

There are several obvious limitations in the current study. One was the relationship between FIGN expression and the prognostic values were analyzed on the public databases, well designed and intensive study should be carried out to testify the results with clinical samples; The other was lacking of investigation on the fine mechanism of the function of FIGN in regulating immune infiltration in HCC, and extra *in vivo* and *in vitro* experiments should be performed to explore it.

In summary, FIGN was considered a predictor of survival biomarker in HCC patients. FIGN expression was correlated with tumor immune cells infiltration, hot tumor classification and thereby enhancing immunotherapeutic efficacy. Findings above will provide brand-new insights for the development of biomarker and immunotherapy for HCC treatment.

## Supplementary Material

Supplementary Materials

Supplementary Figures

Supplementary Tables
