# Supplementary material for: Comprehensive characterization of Fidgetin on tumor immune microenvironment evaluation and immunotherapy in human hepatocellular carcinoma
Source: Aging (Albany NY). 2024 Feb 27;16(5):4445–68. doi: 10.18632/aging.205598 (PMC10968695; doi:10.18632/aging.205598)
Supplement: Supplementary Materials [file aging-16-205598-s001.pdf]

SUPPLEMENTARY MATERIALS

Supplementary Materials and Methods

Quantitative reverse transcription polymerase chain reaction (qRT-PCR)

The HCC and corresponding liver tissues were extracted with TRIzol reagent (Vazyme, Nanjing, China) according to instructions from manufacturer. The purified RNA was quantified and reverse-transcribed with PrimeScript™ RT Master Mix (Takara, Shiga Prefecture, Japan, PR036A). qRT-PCR was carried out with SYBR Premix Ex Taq (Tli RNaseH Plus) (Takara, DRR420A). The synthetized cDNA was taken as template for further amplification by ABI 7500 system. Data in triplicates were collected and normalized to endogenous β-actin (*ACTB*) expression. Primers for *FIGN* and *ACTB* were designed by Pick Primers software (National Center for Biotechnology Information, MD, USA) and listed as follows. *FIGN* forward primer: CGCGTTCAGGCTTGAAGATGC, reverse primer: AACTTTGTGGGCAGGAGACC; *ACTB* forward primer: CGCCGCCAGCTCACC, reverse primer: CACGATGGAGGGGAAGACG. Expression of FIGN was defined as fold change compared to threshold cycle (Ct), and relative expression levels were calculated using the $2^{-\Delta\Delta Ct}$ method by normalization to the housekeeping gene *ACTB*. Results were collected as a means of three individual experiments.

Western Blot

Protein was isolated form HCC and corresponding liver tissues and then normalized to the same concentration with BCA protein assay kit (Thermo Fisher Scientific, Waltham MA, USA). Totally, 60 μg of denatured protein was mounted to 10% SDS-PAGE (Epizyme, Shanghai, China) and transferred to polyvinylidene difluoride (PVDF) membranes (Millipore, Billerica, MA, USA). After blocking with 5% skimmed milk solution for 1 hour in room temperature and incubated with anti-Fidegtin (sc-514956) or anti-β-actin (ab-6276) at 4° C overnight. Extra antibodies were removed by TBST wash and incubated with HRP conjugated secondary antibodies. The blots were visualized with ECL Super Signal (Pierce, Rockford, IL, USA). Images were harvested with Tanon 5200system (Shanghai, China) and processed by ImageJ software (NIH, NY, USA), taking β-actin as an internal control.

Immunohistochemistry

The HCC specimens were fixed in 4% PFA solution and dehydrated by series of alcohol. After transparent in xylene, samples were soaked in wax and then embedded in paraffin. 5μM sections were prepared and undergone dewax and rehydration. Sections went through antigen retrieving, endogenous peroxidase quenching and goat serum incubation. FIGN antibody (sc-514956, 1:150 dilution) was incubated on samples at 4° C overnight. After secondary antibody incubation, the slides were counterstained with hematoxylin. Pictures were taken by Olympus DP71 system and processed by Photoshop software. Each sample was assigned into low, medium and high differentiated group according to Edmonsn-Steiner criteria.
