# Supplementary material for: Comprehensive characterization of Fidgetin on tumor immune microenvironment evaluation and immunotherapy in human hepatocellular carcinoma
Source: Aging (Albany NY). 2024 Feb 27;16(5):4445–68. doi: 10.18632/aging.205598 (PMC10968695; doi:10.18632/aging.205598)
Supplement: Supplementary Figures [file aging-16-205598-s002.pdf]

SUPPLEMENTARY FIGURES

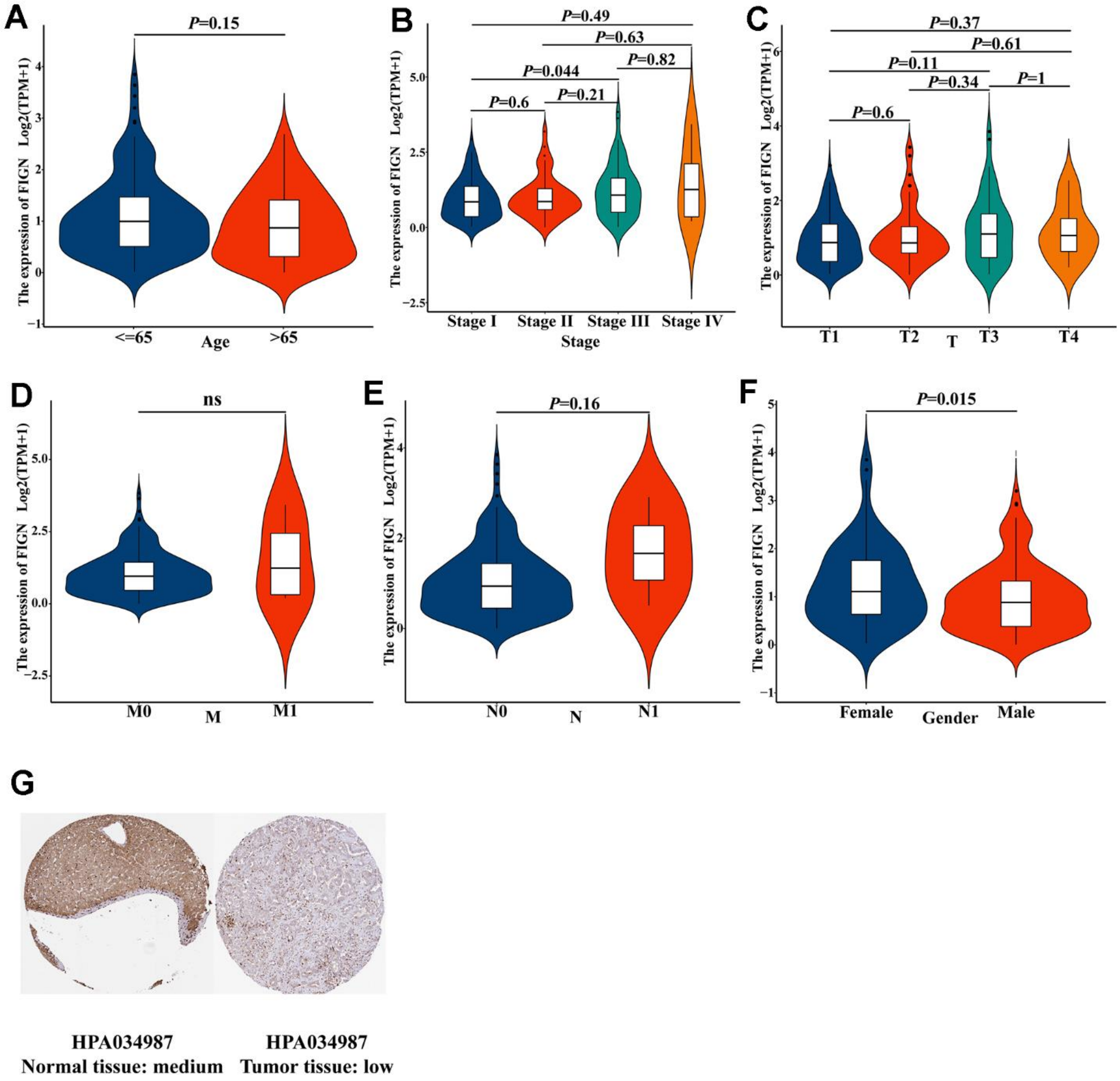

Supplementary Figure 1. Relationship between FIGN and clinicopathological parameters in HCC patients. The violin plots were generated by "ggplot2" package of R software to demonstrate the relationship between *FIGN* mRNA expression and the patient characteristics of age (**A**), stage (**B**), T stage (**C**), M stage (**D**), N stage (**E**), gender (**F**), FIGN expression from HPA database (**G**).

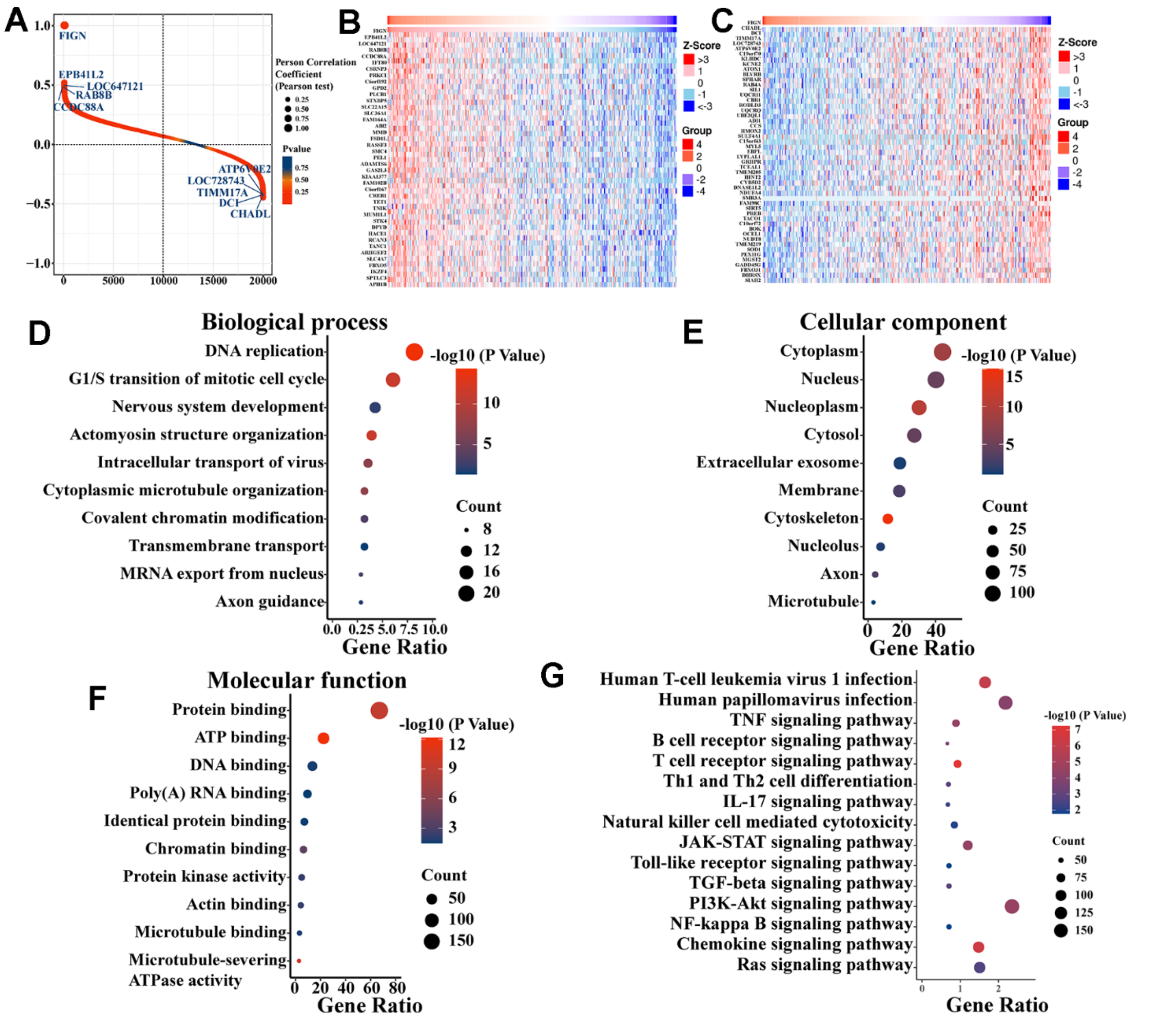

Supplementary Figure 2. Enrichment analysis of FIGN gene co-expression network in HCC. (**A**) Lists of FIGN co-expression genes in TCGA HCC data sets were demonstrated by volcano map. Heat maps illustrated the top 50 co-expression genes positively (**B**) and negatively (**C**) correlated with FIGN expression in HCC data sets. Enrichment analysis of gene ontology (GO) terms for FIGN co-expression genes, as labelled with biological process (**D**), cellular component (**E**), and molecular function (**F**). (**G**) Enrichment analysis of Kyoto Encyclopedia of Genes and Genomes (KEGG) pathway for terms for FIGN co-expression genes.

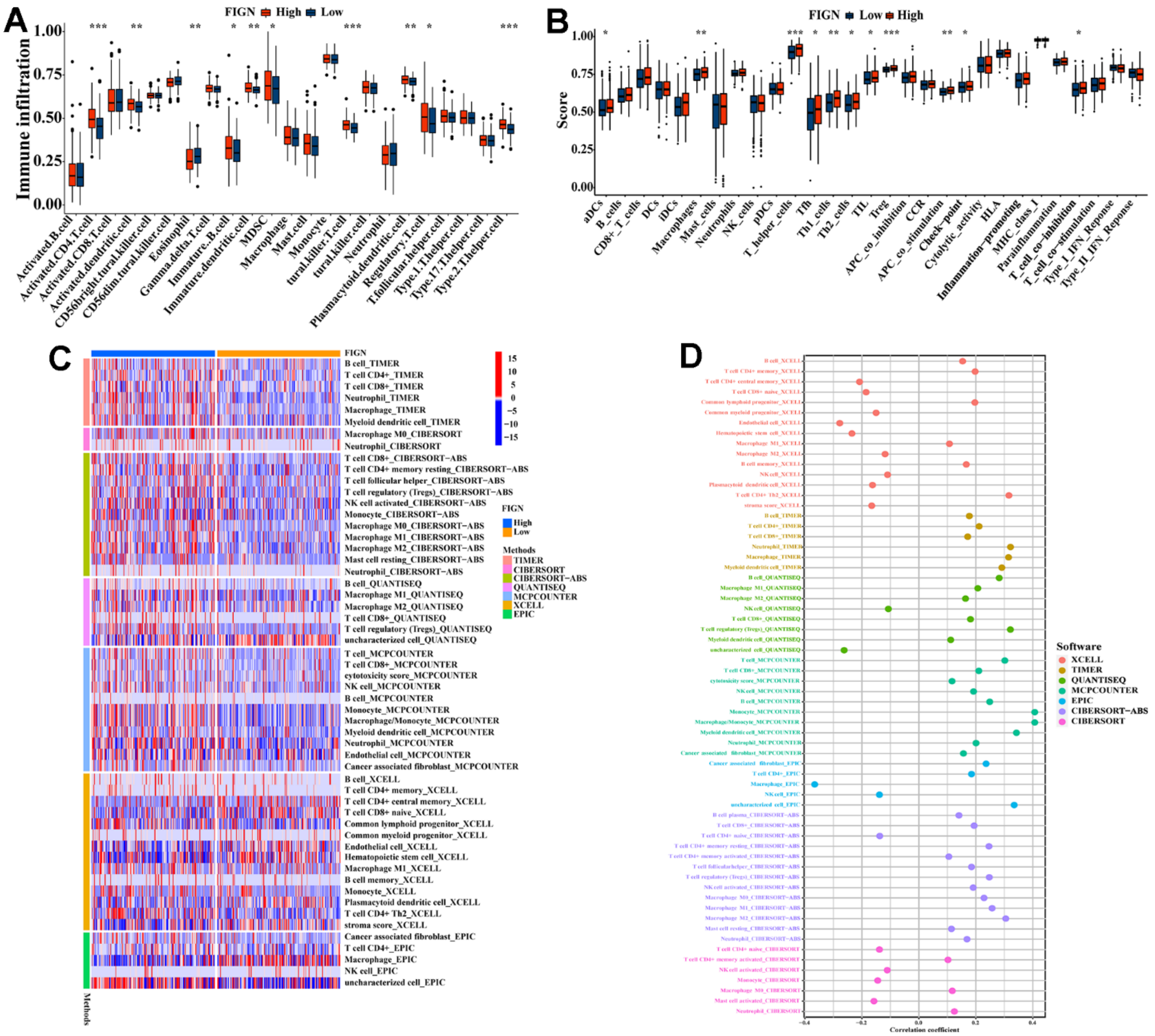

Supplementary Figure 3. Expression of FIGN correlated with tumor infiltrating immune cells. (**A**) Abundance of 23 infiltrating immune cell type between low- or high-FIGN expression groups. (**B**) Differences in immune scores between low- or high-FIGN expression groups. (**C**) Heatmap for immune responses based on TIMER, CIBERSORT, CIBERSORT-ABS, QUANTISEQ, MCPCOUNTER, XCELL, and EPIC algorithms between the low- or high-FIGN expression groups. (**D**) Immune cell bubble diagram illustrated the association of different immune cells with FIGN expression based on different platforms.

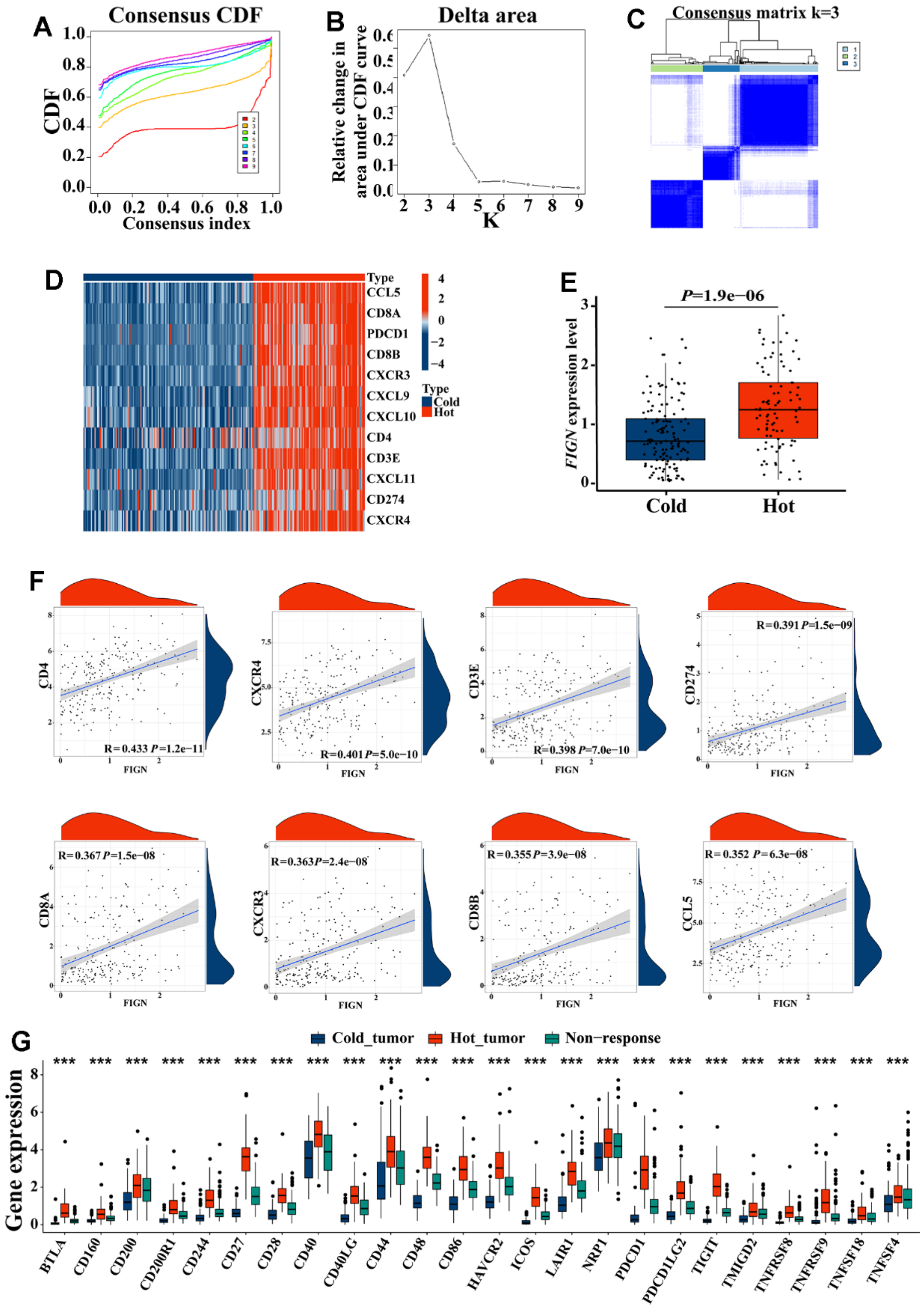

Supplementary Figure 4. FIGN was correlated with hot tumor state and enhanced the response to immunotherapy. (**A**) Consensus cumulative distribution functions (CDF) for the K values (labeled by different colors). (**B**) Relative change in area under the CDF curve illustrated by Delta area plot. (**C**) Consensus matrix of the TCGA-LIHC cohort for k=3. (**D**) Heatmap plot showed hot tumor signature genes were enriched in hot tumor samples. (**E**) FIGN was significantly overexpressed in hot tumors, suggesting it was implicated in therapeutic response to immunotherapy. (**F**) FIGN was critically correlated with multiple predictors of response to immunotherapy, including CD4, CXCR4, CD3E, CD274, CD8A, CXCR3, CD8B, and CCl5 (Spearman's correlation test). (**G**) Expression levels of immune checkpoint genes among hot, cold and non-response tumors in HCC patients.
