# Supplementary material for: Comprehensive characterization of Fidgetin on tumor immune microenvironment evaluation and immunotherapy in human hepatocellular carcinoma
Source: Aging (Albany NY). 2024 Feb 27;16(5):4445–68. doi: 10.18632/aging.205598 (PMC10968695; doi:10.18632/aging.205598)
Supplement: Supplementary Tables [file aging-16-205598-s003.pdf]

SUPPLEMENTARY TABLES

Supplementary Table 1. Clinical characteristics of the hepatocellular carcinoma patients.

Characteristics	Total (N=339)	*FIGN* expression		*P*-value
		High (N=196)	Low (N=143)	
**Age (year)**				0.536
< 65	208 (61.4%)	123 (62.8%)	85 (59.4%)	
≥ 65	131 (38.6%)	73 (37.2%)	58 (40.6%)	
**Gender**				0.074
Male	231 (68.1%)	126 (64.3%)	105 (73.4%)	
Female	108 (31.9%)	70 (35.7%)	38 (26.6%)	
**Family history of cancer**				0.671
NO	196 (57.8%)	116 (59.2%)	80 (55.9%)	
YES	98 (28.9%)	53 (27.0%)	45 (31.5%)	
Unknown	45 (13.3%)	27 (13.8%)	18 (12.6%)	
**TNM stage**				0.928
I	170 (50.1%)	98 (50.0%)	72 (50.3%)	
II	84 (24.8%)	47 (24.0%)	37 (25.9%)	
III	81 (23.9%)	49 (25.0%)	32 (22.4%)	
IV	4 (1.2%)	2 (1.0%)	2 (1.4%)	
**Histologic grade**				0.431
G1–G2	212 (62.5%)	120 (61.2%)	92 (64.3%)	
G3–G4	125 (36.9%)	74 (37.8%)	51 (35.7%)	
Unknown	2 (0.6%)	2 (1.0%)	0 (0%)	
**Ishak score**				**<0.001**
0-4	124 (36.6%)	63 (32.1%)	61 (42.7%)	
5-6	74 (21.8%)	33 (16.8%)	41 (28.7%)	
Unknown	141 (41.6%)	100 (51.0%)	41 (28.7%)	
**Child–Pugh grade**				**0.019**
A	207 (61.1%)	110 (56.1%)	97 (67.8%)	
B-C	21 (6.2%)	10 (5.1%)	11 (7.7%)	
Unknown	111 (32.7%)	76 (38.8%)	35 (24.5%)	
**Vascular invasion**				0.113
None	193 (56.9%)	104 (53.1%)	89 (62.2%)	
Micro	84 (24.8%)	48 (24.5%)	36 (25.2%)	
Macro	14 (4.1%)	9 (4.6%)	5 (3.5%)	
Unknown	48 (14.2%)	35 (17.9%)	13 (9.1%)	
**Alpha fetoprotein**				**0.008**
Negative	143 (42.2%)	69 (35.2%)	74 (51.7%)	
Positive	120 (35.4%)	76 (38.8%)	44 (30.8%)	
Unknown	76 (22.4%)	51 (26.0%)	25 (17.5%)	
**Residual tumor**				0.234
R0	301 (88.8%)	171 (87.2%)	130 (90.9%)	
R1-R2	12 (3.5%)	6 (3.1%)	6 (4.2%)	
Unknown	26 (7.7%)	19 (9.7%)	7 (4.9%)	

**Living status**				**0.130**
Alive	224 (66.1%)	123 (62.8%)	101 (70.6%)	
Dead	115 (33.9%)	73 (37.2%)	42 (29.4%)	
**Disease status**				0.432
NO	163 (48.1%)	97 (49.5%)	66 (46.2%)	
YES	132 (38.9%)	71 (36.2%)	61 (42.7%)	
Unknown	44 (13.0%)	28 (14.3%)	16 (11.2%)	

TNM: Tumor-Node-Metastasis.

Supplementary Table 2. Correlation analysis between FIGN and immune cell marker gene in TIMER, GEPIA2 and TCGA.

**Description**	**Gene markers**	**TIMER**		**GEPIA2**		**TCGA**	
		**Purity**		**Tumor**		**Tumor**	
		**rho**	***P***	**rho**	***P***	**rho**	***P***
	CD19	0.169	1.56e-03	0.220	2.40e-07	0.245	3.40e-07
B cell	MS4A1	0.215	5.59e-05	0.370	2.80e-18	0.245	3.30e-07
	CD79A	0.197	2.28e-04	0.160	2.20e-04	0.236	8.70e-07
	CD8A	0.255	1.60e-06	0.110	1.40e-02	0.250	2.00e-07
$CD8^+$ T Cell	CD8B	0.203	1.44e-04	0.075	8.60e-02	0.235	9.90e-07
	IL2RA	0.308	4.67e-09	-0.003	9.50e-01	0.241	5.30e-07
	CXCR3	0.253	1.79e-06	-0.063	1.50e-01	0.262	4.90e-08
Tfh	CXCR5	0.220	3.54e-05	0.038	3.90e-01	0.230	1.60e-06
	ICOS	0.322	8.33e-10	0.140	1.60e-03	0.307	1.10e-10
	IL12RB1	0.297	1.75e-08	0.093	3.20e-02	0.266	3.00e-08
Th1	CCR1	0.378	3.34e-13	0.087	4.50e-02	0.312	6.40e-11
	CCR5	0.358	6.53e-12	0.110	1.40e-02	0.331	2.70e-12
	CCR4	0.397	1.75e-14	0.210	6.20e-07	0.361	1.80e-14
Th2	CCR8	0.425	1.24e-16	0.120	7.80e-03	0.320	1.40e-11
	HAVCR1	0.399	1.28e-14	0.200	2.40e-06	0.345	2.70e-13
	IL21R	0.349	2.38e-11	0.210	2.50e-01	0.318	2.80e-11
Th17	IL23R	0.340	7.91e-11	0.250	8.30e-09	0.330	3.30e-12
	CCR6	0.368	1.60e-12	0.008	8.50e-01	0.399	<2.2e-16
	FOXP3	0.285	6.65e-08	0.044	3.20e-01	0.171	4.20e-04
Treg	NT5E	0.257	1.28e-06	0.110	1.10e-02	0.246	3.10e-07
	IL7R	0.388	7.33e-14	0.240	2.60e-08	0.275	9.60e-09
	PDCD1	0.268	4.13e-07	0.096	2.80e-02	0.278	5.60e-09
T cell exhaustion	CTLA4	0.260	9.29e-07	0.068	1.20e-01	0.273	1.10e-08
	LAG3	0.254	1.65e-06	0.310	1.20e-13	0.278	7.00e-09
	NOS2	0.071	1.85e-01	-0.210	8.70e-07	0.025	6.10e-01
M1 Macrophage	IRF5	0.273	2.49e-07	0.140	1.70e-03	0.211	1.20e-05
	PTGS2	0.303	9.06e-09	0.400	2.30e-21	0.260	5.60e-08
	CD163	0.274	2.36e-07	0.081	6.30e-02	0.232	1.50e-06
M2 Macrophage	MRC1	0.170	1.50e-03	0.180	2.60e-05	0.138	4.40e-03
	CD209	0.246	3.62e-06	0.004	9.20e-01	0.206	2.00e-05
	CCL2	0.195	2.62e-04	0.120	4.70e-03	0.161	8.70e-04
TAM	CD86	0.329	3.68e-10	-0.011	8.00e-01	0.300	3.40e-10
	CD68	0.254	1.72e-06	-0.036	4.10e-01	0.193	6.70e-05
Monocyte	CD14	-0.097	7.28e-02	-0.210	2.00e-06	-0.122	1.20e-02

	CD33	0.272	2.84e-07	0.240	1.80e-08	0.285	2.60e-09
	ITGAX	0.344	4.49e-11	0.360	2.20e-17	0.272	1.50e-08
	B3GAT1	0.111	3.93e-02	0.280	6.50e-11	0.124	1.10e-02
Natural killer cell	KIR3DL1	0.145	7.07e-03	0.160	2.60e-04	0.125	9.70e-03
	CD7	0.240	6.60e-06	0.160	2.40e-04	0.276	8.90e-09
	FCGR3A	0.327	4.45e-10	0.140	1.20e-03	0.279	6.30e-09
Neutrophil	CD55	0.114	3.46e-02	-0.030	4.90e-01	0.009	8.60e-01
	ITGAM	0.311	3.39e-09	0.290	8.40e-12	0.285	2.60e-09
	CD1C	0.254	1.65e-06	-0.003	9.60e-01	0.272	1.30e-08
Dendritic cell	THBD	0.178	8.84e-04	0.130	2.00e-03	0.122	1.20e-02
	NRP1	0.345	4.42e-11	0.016	7.20e-01	0.233	1.30e-06
